# Does body image perception relate to quality of life in middle-aged women?

**DOI:** 10.1371/journal.pone.0184031

**Published:** 2017-09-19

**Authors:** Maria Socorro Medeiros de Morais, Rafaela Andrade do Nascimento, Mariana Carmem Apolinário Vieira, Mayle Andrade Moreira, Saionara Maria Aires da Câmara, Álvaro Campos Cavalcanti Maciel, Maria das Graças Almeida

**Affiliations:** 1 Health Sciences Center of Federal University of Rio Grande do Norte, Natal, RN, Brazil; 2 Physiotherapy Department of Federal University of Rio Grande do Norte, Natal, RN, Brazil; 3 Physiotherapy Department of Federal University of Ceará, Fortaleza, CE, Brazil; 4 Physiotherapy Department of Federal University of Rio Grande do Norte, Santa Cruz, RN, Brazil; University of Zurich, SWITZERLAND

## Abstract

**Objective:**

In Brazil, information about the influence of body image on the various life domains of women in menopausal transition is scarce. Thus, the objective of the study was to analyze the relationship between body image and quality of life in middle-aged Brazilian women.

**Methods:**

This was a cross-sectional study of 250 women between 40 and 65 years old, living in Parnamirim/RN, Brazil, who were evaluated in relation to body image and quality of life. For body image, women were classified as: dissatisfied due to low weight, satisfied (with their body weight) and dissatisfied due to being overweight. Quality of life was assessed through a questionnaire in which higher values indicate higher quality of life. Multiple linear regression was performed to analyze the relationship between body image and quality of life, adjusted for covariates that presented p<0.20 in the bivariate analysis.

**Results:**

The average age was 52.1 (± 5.6) years, 82% of the women reported being dissatisfied due to being overweight, and 4.4% were dissatisfied due to having low weight. After multiple linear regression analyzes, body image remained associated with health (p<0.001), emotional (p = 0.016), and sexual (p = 0.048) domains of quality of life, as well as total score of the questionnaire (p<0.001).

**Conclusion:**

Women who reported being dissatisfied with their body image due to having low weight or overweight had worse quality of life in comparison to those who were satisfied (with their body weight).

## Introduction

Body image is defined as a subjective satisfaction with the one’s body [1]. The body image construct is multidimensional and focused on the weight, shape and degree to which individuals are satisfied with their appearance [2]. Moreover, body image varies according to gender. Men (tend to) prefer muscular bodies, while women wish to be lean/thin, showing greater dissatisfaction when they perceive their body as bigger than the desired size [3].

Additionally, during the female aging process, women experience a remarkable physiological period in the middle-age phase, known as climacteric, which is characterized by the progressive loss of ovarian function and hormonal changes that signal passage from the reproductive to the non-reproductive phase [4]. Evidence suggests that during this period, there is a particular decrease in estradiol, which leads to an increase in fat mass, with modifications in its distribution pattern [5,6]. A greater accumulation of adipocytes goes from the femur-gluteal region and subcutaneous tissue (gynoid pattern) to being deposited more into the abdomen region (android pattern) [7], leading to a negative effect on the health of middle-aged women [4].

In addition to hormonal factors, the North American Menopause Society (NAMS) states that in, lifestyle and behavioral factors in female aging, such as lack of exercise and increased consumption of unhealthy foods, are also closely linked to the weight gain that occurs in that period [8]. This inassociation to other factors, such as the multiple climacteric symptoms, makes the menopausal transition a period in which most women do not positively cope with, causing greater dissatisfaction with their body, with the aging process and with their body image [9].

The physical changes that occur with the aging process may cause a modification of one´s own image, leading to unconformity between the desired and the real image. This perception can have important consequences on their health and quality of life [10], being associated with conditions such as depression, eating disorders [11], low self-esteem and self-confidence [12]. Moreover, dissatisfaction with body image can negatively influence social interaction, job opportunities, productivity, socioeconomic status, and psychosocial performance [13].

Although there are studies on this subject, most investigate body image in a population of adolescents and young adult women. It was observed that body image was associated with impairment of quality of life relating to some physical and mental aspects in women aged 18 to 42 [14]. However, there are few studies conducted on the other age groups, especially in relation to aging and body changes that occur in middle-aged women [15]. Due to the impact of body image changes on women's lives in climacteric, understanding body perception is critical to the healthcare of this population. Thus, the objective of the study is to analyze the relationship between body image and quality of life in middle-aged women from Northeast Brazil.

## Material and methods

This is an observational, analytical cross-sectional study. In this article, data collected between September 2014 and July 2015 were used from an ongoing longitudinal study that analyzes the influence of menopause on sarcopenia and physical performance in middle-aged women [16,17,18].

### Population and sample

The study population was composed of 40 to 65 year-old women living in Parnamirim, a city in Northeast Brazil, located in the metropolitan region of the capital of Rio Grande do Norte state, called Natal [17]. The sample of the baseline study was composed of 500 women recruited during April to November of 2013 by advertisements in the primary care neighborhood centers across the city. As exclusion criteria for this baseline data collection, women could not present neurological diseases such as Parkinson's, stroke or any condition that might compromise the evaluation of the physical function measures; being a smoker; or having had double oophorectomy. Follow up data from the longitudinal study were used for the present article, in which 250 women were evaluated regarding their body image. The overall characteristics of the sample were not different from baseline (S1 Appendix).

### Ethics

The present study was approved by the Research Ethics Committee of the Federal University of Rio Grande do Norte, opinion number 387.737, and all the volunteers signed the Clear and Informed Consent Form being in agreement with resolution 510/2016 of the National Health Council.

### Procedures

All the women were evaluated by trained interviewers using a standardized questionnaire at the Integrated Nucleus of Education, Research and Community Actions located in the city of Parnamirim—RN, as described below.

Sociodemographic data such as age, education level and family income were collected. Education level was categorized into: up to Basic Education (0 to 7 years), between Basic Education and Secondary Education (8 to 11 years), and Secondary Education or more (12 years or more) [17]. Family income was categorized using the Brazilian minimum wage (MW), which can be defined as the lowest remuneration that employers may legally pay workers per month. At the time of the interview, the MW was set at 788.00BRL (seven hundred and eighty-eight Brazilian Reals), equivalent to around 246.00USD (two hundred and forty six US Dollars). Family income was dichotomized as less than 3 MW, and 3 MW or more [17].

Menopausal stages were determined using the STRAW classification stages [19], being classified into three groups according to self-reported menstruation pattern: pre-menopause (regular menses), perimenopause (irregular menstruation, with difference in cycle length greater than seven days up to one year of amenorrhea) or postmenopause (absence of menstruation for more than one year).

Regarding reproductive history, three variables were considered: age at menarche, age at first birth and parity. For age at menarche, they were classified as before of age 13, at age 13, and after age 13, because more than one fourth of women reported their menarche was at age 13. Maternal age at the first child was dichotomized before 18 years and after 18 years, with the intention of separating women who gave birth in adolescence from the others [16]. Parity was collected through participants’ self-report, which was categorized into less than 3 children, and 3 children or more [16]. This cut-off was selected based on evidence that having three or more children is associated to long term adverse health outcomes [20].

In order to evaluate the presence of associated comorbidities, questions from the Women’s Health on Aging Study [21] were used, asking women if any doctor or health professional had already stated that they had the following conditions: systemic arterial hypertension, diabetes mellitus or depression.

The Utian Quality of Life Questionnaire (UQol) was used to evaluate the self-perceived quality of life. This instrument has been adapted for use in Brazil with high reliability (Cronbach's α = 0.82) and good construct validity [22]. This questionnaire contains 23 questions relating to four domains: occupational, health, sexual, and emotional; and it uses a Likert-like scale with five options ranging from ‘*1-not true for me’* to *‘5-very true for me’* for each question. The total and domain scores were computed, from the responses being defined as the sum of scores, and with the higher score being indicative of better quality of life [22].

Among the various instruments that are used to evaluate body image, the Stunkard scale has been widely used by epidemiological studies for being practical, quick and easy to apply [23] This scale consists of drawings with different human forms numbered from 1 to 9, where the first silhouette/image is the leanest and the ninth is the most obese. The women chose two silhouettes from a set presented to them; one that best represented their current physical appearance, and the one they would like to have. The scale score was given by the result of the difference in the number obtained between the current body silhouette and the desired silhouette. Thus, a subject who scores zero is considered satisfied with their body image, while any other scores indicates dissatisfaction with their personal body image [23]. When the difference is positive, it represents dissatisfaction due to being overweight; and when it is negative, it refers to dissatisfaction due to low weight/being too thin [24]. The current body image of this scale showed a good correlation to the body mass index when applied to a Brazilian population [25]. In addition, greater dissatisfaction was found among those with eating disorders than those without it, showing that this is a valid scale to evaluate body image among Brazilian women [25].

### Statistical analysis

Data were analyzed using the SPSS version 20.0 statistical software (SPSS, Chicago, IL, USA). A descriptive analysis of the sample was initially performed using means and standard deviations for quantitative variables, and absolute and relative frequencies for the categorical variables. The relation between quality of life (domains and total) and categorical variables was evaluated using Student’s t-test or ANOVA with Tukey Post Hoc, according to the number of categories of the variables. Next, a multiple linear regression analysis was performed for the UQol domains (occupational, health, emotional and sexual), and the total score adjusted by the covariates, which presented p<0.20 in the bivariate analysis, with only the statistically significant variables remaining in the final models. A significance level or p value <0.05 and 95% confidence intervals were used for all tests.

## Results

The sample of the present study was composed of 250 women with mean of age of 52.1 (± 5.6) years, of which 46.5% reported having an education level between Basic and Secondary Education. Regarding income, 69.0% reported receiving less than 3 minimum wages. In relation to body image (Stunkard Scale), 4.4% of the women reported being dissatisfied due to low weight/being thin, while 82% of the women were dissatisfied due to being overweight. Other characteristics of the sample are described in Table 1.

**Table 1:** Characterization of the sample.

Variables		Mean (±SD) or n (%) *	
Age		52.1	(5.6)
Education level	Up to Basic Education	102	(40.9)
	Between Basic and Secondary Education	116	(46.5)
	Secondary Education or more	31	(12.4)
Stable union	No	67	(26.8)
	Yes	183	(73.2)
Ethnicity	White	88	(35.2)
	Brown	150	(60.0)
	Black	12	(4.8)
Family income	≥ 3 MW	77	(30.9)
	< 3 MW	172	(69.0)
Menopausal stage	Pre-menopause	57	(29.3)
	Perimenopause	40	(20.6)
	Postmenopause	97	(50.0)
Age at menarche	Before the age of 13	92	(36.8)
	At the age of 13	63	(25.2)
	After the age of 13	95	(38.0)
Age at first birth	No children	7	(2.8)
	< 18 years	55	(22.0)
	≥ 18 years	188	(75.2)
Parity	0 to 2 children	114	(45.6)
	3 children or more	136	(54.4)
Hypertension	No	118	(47.2)
	Yes	132	(52.8)
Diabetes	No	221	(88.4)
	Yes	29	(11.6)
Depression	No	201	(80.4)
	Yes	49	(19.6)
Body image	Dissatisfied due to low weight (<0)	11	(4.4)
	Satisfied (0)	34	(13.6)
	Dissatisfied due to overweight (>0)	205	(82.0)

* n valid

Table 2 shows the results of quality of life mean comparison in relation to body image and other covariates. Lower scores for the health and emotional domains of quality of life, as well as for the total score, were found for those referring dissatisfaction with their body image. Regarding the age variable in relation to the quality of life domains, the correlation results were as follows: occupational UQoL (r = 0.01, p = 0.81) health UQoL (r = 0.11, p = 0.07), emotional UQoL (r = 0.01, p = 0.79), sexual UQoL (r = -0.04, p = 0.48) and total UQoL (r = 0.05, p = 0.42). All correlations were positive, with the exception of the sexual domain. Therefore, the greater the age, the higher the score indicates better quality of life, while. The opposite occurs, in the sexual domain.

**Table 2:** Relationship between independent variables and the quality of life domains.

VARIABLES (n = 250)	Occupational UQoL	Health UQoL	Emotional UQoL	Sexual UQoL	Total UQoL
**Education level**	p = 0.47	p = 0.25	**p = 0.04**^**a**^	**p = 0.04**^**b**^	p = 0.76
Up to Basic Education	25.5 (4.1)	19.7 (4.0)	20.2 (3.2)	8.9 (2.6)	74. 5 (9.7)
Between Basic and Secondary Education	25.6 (4.0)	19.6 (4.6)	20.9 (2.9)	9.1 (2.5)	75.3 (9.9)
Secondary Education or more	26.6 (4.8)	18.2 (5.6)	21.6 (2.6)	7.7 (2.9)	74.2 (12.2)
**Stable union**	p = 0.35	p = 0.76	p = 0.52	**p = 0.03**	p = 0.87
Yes	25.5 (4.1)	19.4 (4.6)	20.7 (3.1)	9.1 (2.5)	74.8 (10.4)
No	26.1 (4.1)	19.6 (4.4)	20.9 (2.9)	8.3 (2.8)	75.0 (9.2)
**Ethnicity**	p = 0.28	p = 0.61	p = 0.57	p = 0.37	p = 0.30
White	25.6 (4.5)	19.7 (4.4)	20.7 (3.2)	8.6 (2.7)	74.5 (11.3)
Brown	25.7 (4.0)	19.3 (4.7)	20.8 (3.0)	9.0 (2.7)	74.8 (9.6)
Black	27.6 (2.3)	20.5 (3.5)	21.7 (3.0)	9.6 (2.4)	79.3 (6.4)
**Family income**	p = 0.16	p = 0.96	**p = 0.03**	p = 0.73	p = 0.17
≥ 3 MW	26.3 (4.0)	19.5 (4.9)	21.4 (2.8)	8.9 (2.9)	76.2 (10.3)
< 3 MW	25.5 (4.2)	19.5 (4.3)	20.5 (3.1)	8.8 (2.6)	74.3 (10.0)
**Menopausal stage**	p = 0.15	p = 0.27	p = 0.07	p = 0.06	p = 0.25
Premenopause	25.2 (4.4)	19.2 (4.2)	20.2 (2.8)	8.5 (2.9)	73.1 (10.4)
Perimenopause	26.7 (3.0)	18.4 (3.9)	21.6 (2.9)	9.7 (2.3)	76.5 (8.9)
Postmenopause	25. (4.1)	19.8 (4.7)	20.9 (3.2)	8.7 (2.6)	74.8 (9.8)
**Age at menarche**	p = 0.21	p = 0.30	p = 0.27	p = 0.85	p = 0.20
Before the age of 13	25.6 (4.0)	19.0 (4.3)	20.9 (2.9)	8.8 (2.7)	74.4 (9.3)
At the age of 13	25.1 (4.2)	19.4 (4.7)	20.2 (3.2)	8.8 (2.9)	73.5 (10.9)
After the age of 13	26.3 (4.1)	20.0 (4.7)	21.0 (3.2)	9.0 (2.5)	76.3 (10.3)
**Parity**	p = 0.19	p = 0.81	p = 0.11	p = 0.19	p = 0.77
0 to 2 children	25.4 (4.4)	19.6 (4.7)	21.1 (3.0)	8.6 (2.6)	74.7 (10.4)
3 children or more	26.0 (3.9)	19.4 (4.5)	20.5 (3.1)	9.0 (2.7)	75.0 (9.9)
**Age at first birth**	p = 0.21	p = 0.64	p = 0.12	p = 0.95	p = 0.45
No children	23.1 (4.9)	20.4 (5.3)	20.0 (3.9)	8.7 (2.7)	72.3 (12.0)
< 18 years	25.6 (3.9)	19.0 (4.5)	20.0 (3.3)	8.9 (2.8)	73.7 (10.2)
≥ 18 years	25.9 (4.1)	19.6 (4.5)	21.0 (2.9)	8.9 (2.6)	75.4 (10.0)
**Hypertension**	p = 0.48	p = 0.08	p = 0.12	p = 0.48	p = 0.08
Yes	25.6 (4.2)	19.0 (4.5)	20.5 (3.1)	8.8 (2.7)	73.9 (9.8)
No	25.9 (4.1)	20.0 (4.6)	21.2 (3.0)	9.0 (2.6)	76.0 (10.4)
**Diabetes**	p = 0.91	**p = 0.02**	**p = 0.03**	p = 0.51	p = 0.16
Yes	25.6 (5.2)	21.3 (3.9)	21.9 (3.2)	8.6 (2.9)	77.4 (10.9)
No	25.7 (3.9)	19.3 (4.6)	20.6 (3.0)	8.9 (2.6)	74.6 (10.0)
**Depression**	p = 0.07	**p = 0.01**	**p < 0.001**	p = 0.07	**p <0.001**
Yes	24.8 (4.0)	18.0 (4.6)	18.4 (3.0)	8.3 (2.5)	69.5 (9.8)
No	25.9 (4.1)	19.9 (4.5)	21.3 (2.8)	9.0 (2.7)	76.2 (9.8)
**Body image**	p = 0.13	**p <0.001**^**c**^	**p = 0.03**^**d**^	p = 0.09	**p = 0.002**^**e**^
Dissatisfied due to low weight (< 0)	23.3 (3.7)	19.5 (3.7)	19.4 (3.4)	8.7 (2.5)	70.9 (9.3)
Satisfied (= 0)	25.8 (4.3)	22.7 (3.8)	21.8 (2.9)	9.8 (2.6)	80.2 (8.0)
Dissatisfied due to overweight (> 0)	25.8 (4.1)	18.9 (4.5)	20.7 (3.0)	8.7 (2.7)	74.2 (10.2)

a: ≠ Between Basic Education x Secondary Education or more

b: ≠ Between Basic and Secondary Education x Secondary Education or more

c: ≠ Between Satisfied x Dissatisfied due to overweight

d: ≠ Between Satisfied x Dissatisfied due to low weight

e: ≠ Between Satisfied x Dissatisfied due to low weight and between Satisfied x Dissatisfied due to overweight

Table 3 shows the linear regression analysis results for the quality of life outcome (domains and total UQoL). Body image remained statistically related to all domains, as well as to the total score, only not being related to the occupational domain. Compared to those satisfied to their body image, those dissatisfied due to having low weight present significantly lower scores in the health (less 3 points) and emotional (less two points) domains, and to for the total score of UQoL (less almost 8 points). Those dissatisfied with being overweight presented lower UQoL scores for health, emotional and sexual domains, as well as for the total score, compared to those satisfied to their body image. There is no relation between body satisfaction and the occupational domain of UQoL in the linear regression models (dissatisfied due to having low weight β = -2,462, p = 0,06 and dissatisfied with being overweight β = -0,094, p = 0,90).

**Table 3:** Multiple linear regression analysis for the UQoL domains and total score. Parnamirim, RN, Brazil, 2017.

**UQoL Health Domain**				
Variables		B	95% CI	p value
Constant		17.509	11.548–23.471	<0.001
**Body image**				
Dissatisfied due to having low weight (<0)		-3.077	-6.023–-0.131	0.041
Dissatisfied due to being overweight (>0)		-3.759	-5.302–-2.207	<0.001
Satisfied (= 0)		0		
Adjusted for age, hypertension, diabetes and depression.				
**UQoL Emotional Domain**				
Variables		B	95% CI	p value
Constant		20.795	19.218–22.373	< 0.001
**Body image**				
Dissatisfied due to having low weight (<0)		-2.020	-3.949–-0.090	0.040
Dissatisfied due to being overweight (>0)		-1.263	-2.292–-0.235	0.016
Satisfied = 0		0		
Adjusted for diabetes and depression.				
**UQoL Sexual Domain**				
Variables		B	95% CI	p value
Constant		9.582	8.554–10.611	< 0.001
**Body image**				
Dissatisfied due to having low weight (<0)		-1.316	-3.393–0.761	0.213
Dissatisfied due to being overweight (>0)		-1.047	-2.082–-0.012	0.048
Satisfied (= 0)		0		
Adjusted for menopausal stage.				
**Total UQoL Score**				
Variables		B	95% CI	p value
Constant		77.447	72.077–82.816	<0.001
**Body image**				
Dissatisfied due to having low weight (<0)		-7.690	-14.245–-1.135	0.022
Dissatisfied due being to overweight (>0)		-5.839	-9.333–-2.345	0.001
Satisfied (= 0)		0		
Adjusted for hypertension, diabetes and depression.				

## Discussion

The present study analyzed the relationship between body image and quality of life in middle-aged women in a region of Northeast Brazil. The total score and the quality of life domains had a significant relation to body image (with the exception of the occupational domain), with a better result among women who were satisfied with their image in relation to those who were dissatisfied.

Among the evaluated women, it was found that 82% reported being dissatisfied due to being overweight. The few studies found with middle-aged women on this subject addressed a wide age group, where middle age was considered as a category [26,27]. Changes in body composition due to menopause in middle-aged women alter the pattern of fat distribution [7], interfering with body image [15], which could be related to the high degree of body dissatisfaction due to being overweight rather than having low weight found in the present study. These findings are consistent with the findings of Benkeser et al. (2012) [26], who evaluated 2,814 Ghanaian women aged 18 years or more and found that 76.3% of 35–54 year olds were dissatisfied with their body image and 41.8% of women preferred a smaller figure [26]. Runfola et al. (2013) [27] found a mean of 88.9% of body dissatisfaction for the age groups of 45–54 years and of 89.0% in the group of 55–64 years in their study with 5,869 American women between 25 and 89 years and the majority of women preferred a smaller silhouette than their current silhouette [27].

In this study, we found strong associations between body image and the different domains of quality of life. Body image remained associated to health, emotional, and sexual domains, as well as to the total UQoL score. Body image interferes in several aspects of women's daily lives, such as self-esteem, relationships, and job stability, among others, and it can compromise the role of women in society, with negative consequences on their quality of life [15].

Regarding the health domain, studies report that body dissatisfaction is associated with an increase in the probability of impairment for overall health aspects [3, 15, 28, 29]. People who are dissatisfied with their body are able to promote a negative self-assessment of health and consequently their quality of life [29]. Since 82% of the women were dissatisfied due to being overweight, this relationship with the health domain may reflect in women recognizing an association of being overweight to an unhealthy condition, with consequent worse scores on the evaluation of that domain.

Concerning the UQoL emotional domain in the present study, women who were dissatisfied with their body image presented worse scores when compared to those who were satisfied. In a study by Yazdandoost et al. (2016) [30], people who have greater dissatisfaction with their body image experience more negative emotions, such as anxiety, shame or sadness [30]. Thus, dissatisfaction with one’s own body image can result in low self-confidence, risk of eating disorders and depression [3,31]. Considering the association between the negative perception of body image and commitment to emotional well-being, we can expect that the effects of dissatisfaction with body image interfere in mental and social health, affecting quality of life [32], which corroborates the findings of the present study regarding the emotional domain.

In the present study, we observed that body image remained related to the UQoL sexual domain, in which the worst scores were found in women who were dissatisfied due to being overweight, corroborating the findings by Flynn et al. (2016) [33]. These authors claim that sexual health is an important predictor of a good quality of life [33]. Evidence suggests that a woman's interpretation of their experiences during menopause is related to the quality of sexual experiences and also to their body image [15,34]. Women with negative attitudes perceive the physical changes that occur in this phase in a negative way, such as weight gain and reduced sexual desire, which may interfere with body image, leading these women to feel less sexually desirable and decreasing their quality of life [35].

It has been cited that sociocultural patterns, socioeconomic status, educational level and the media can affect perception of the ideal body [3]. In Brazil, studies with teenagers reported non-association among body dissatisfaction and socioeconomic status, showing that this problem is equal for high, middle or low-income populations in this country [13]. Another recent longitudinal study with Brazilian teenagers showed that body dissatisfaction is even more influenced by sociocultural aspects than to biological or psychological variables [36]. This may be a consequence of the necessity of self-affirmation among peers and the imposition of an ideal beauty pattern in Brazilian society [37]. Dissatisfaction with body image is a reason for public health concern due to the high prevalence, as found in this study, and because it is often related to worse behavioral habits, such as physical inactivity and poor dietary habits [13]. Moreover, as shown in the present study, body dissatisfaction is associated to poorer quality of life, which highlights the need of public health policies addressing these issues.

### Strengths and limitations of the study

We considered the analysis of the relationship between body image and quality of life in middle-aged women as a strength, since studies in this age group are scarce. Based on the recognition of this relationship, it is possible to encourage measures aimed at improving body perception such as regular physical exercise, as well as reducing excess weight, thus seeking to improve the quality of life in women in this age group. In addition, validated scales were used for this population.

Regarding limitations of the study, nutritional status is a factor that seems to be associated with body image, and therefore quality of life, but it was not analyzed. A nutritional intervention could correct or improve the pattern of food consumption and the anthropometric profile, which could result in benefits related to the physical and mental health of these women [38].

The presence or absence of diseases was evaluated through self-reporting by participants regarding medical diagnoses. Collecting self-reported variables can generate bias, especially in a population with low education levels. However, these types of questionnaires are widely used in epidemiological studies. Furthermore, the cross-sectional design limits causal inferences, requiring longitudinal studies on this theme to achieve these objectives. Although the sample composition was by convenience, its representativeness can be assured since sociodemographic data resemble population-based studies conducted in the area [39,40].

## Conclusion

Based on the results found in the analyses, it is possible to conclude that body image is related to quality of life in middle-aged women. Body image was associated with health, emotional and sexual domains of quality of life, as well as with total UQoL score. It is important to note that women dissatisfied with their weight due to low weight represented only 4.4% of the sample, evidencing that the majority of the women dissatisfied with their weight complain about the being overweight, which is common due to changes in body composition in the climacteric period. Considering the high level of body dissatisfaction of these women, it is believed that using valid instruments, the present study is of fundamental importance for improving the profile of female aging, providing scientific subsidies for planning preventive actions in health aimed at the quality of life of this population.

## Supporting information

S1 Appendix (DOCX)
