# Supplementary material for: Does body image perception relate to quality of life in middle-aged women?
Source: PLoS One. 2017 Sep 19;12(9):e0184031. doi: 10.1371/journal.pone.0184031 (PMC5604940; doi:10.1371/journal.pone.0184031)
Supplement: S1 Appendix — (DOCX) [file pone.0184031.s001.docx]

**S1 Appendix: Comparison between this sample and the whole sample (baseline)**

|  | **Baseline sample (N = 497)** | **Actual Sample**  **(N= 250)** |
| --- | --- | --- |
| **Age** | 49.96 (5.6) | 52.00 (5.6) |
| **Family income** |  |  |
| Less than 3MW | 349 (70.2) | 172 (68.8) |
| 3MW or more | 148 (29.8) | 78 (30.2) |
| **Education** |  |  |
| Basic education | 208 (41.9) | 103 (41.2) |
| Between basic and secondary | 206 (41.4) | 116 (46.4) |
| or more | 83 (16.6) | 31 (12.4) |
